# Leptospirosis Outbreaks in Nicaragua: Identifying Critical Areas and Exploring Drivers for Evidence-Based Planning

**DOI:** 10.3390/ijerph9113883

**Published:** 2012-10-26

**Authors:** Maria Cristina Schneider, Patricia Nájera, Sylvain Aldighieri, Jorge Bacallao, Aida Soto, Wilmer Marquiño, Lesbia Altamirano, Carlos Saenz, Jesus Marin, Eduardo Jimenez, Matthew Moynihan, Marcos Espinal

**Affiliations:** 1 Pan American Health Organization, Health Surveillance and Disease Prevention and Control,525 23rd. St. NW, Washington, DC 20037, USA; Email: najerapa@paho.org (P.N.); aldighsy@paho.org (S.A.); mattm929@gmail.com (M.M.); espinalm@paho.org (M.E.); 2 University of Medical Sciences ofHabana, Research and Reference Center of Atherosclerosis of Havana, Universidad de Ciencias Médicas de La Habana, Tulipán y Panorama, Plaza, La Habana, Cuba; Email: bacallao@infomed.sld.cu; 3 Pan American Health Organization Nicaragua, PO Box 1309, Managua, Nicaragua; Email: sotoa@nic.ops-oms.org (A.S.); marquinw@nic.ops-oms.org (W.M.); altamirles@hotmail.com (L.A.); 4 Ministry of Health of Nicaragua, Costado Oeste Colonia Primero de Mayo, PO Box 107, Managua, Nicaragua; Email: carlossaen@gmail.com (C.S.); dgvs@minsa.gob.ni (J.M.); zoonosis@minsa.gob.ni (E.J.)

**Keywords:** leptospirosis, risk, outbreaks, Nicaragua, Central America

## Abstract

Leptospirosis is an epidemic-prone zoonotic disease that occurs worldwide. In Central America, leptospirosis outbreaks have been reported in almost all countries; Nicaragua in particular has faced several outbreaks. The objective of this study was to stratify the risk and identify “critical areas” for leptospirosis outbreaks in Nicaragua, and to perform an exploratory analysis of potential “drivers”. This ecological study includes the entire country (153 municipalities). Cases from 2004 to 2010 were obtained from the country’s health information system, demographic and socioeconomic variables from its Census, and environmental data from external sources. Criteria for risk stratification of leptospirosis were defined. Nicaragua reported 1,980 cases of leptospirosis during this period, with the highest percentage of cases (26.36%) in León, followed by Chinandega (15.35%). Among the 153 municipalities, 48 were considered critical areas, 85 were endemic and 20 silent. Using spatial and statistical analysis, the variable presenting the most evident pattern of association with critical areas defined by top quintile of incidence rate is the percentage of municipal surface occupied by the soil combination of cambisol (over pyroclastic and lava bedrock) and andosol (over a volcanic ashes foundation). Precipitation and percentage of rural population are also associated with critical areas. This methodology and findings could be used for Nicaragua’s Leptospirosis Intersectoral Plan, and to identify possible risk areas in other countries with similar drivers.

## 1. Introduction

Leptospirosis is an epidemic-prone zoonotic disease that occurs all over the World, with the highest incidence found in countries with humid subtropical or tropical climates. The disease affects the most vulnerable populations and is mostly associated with poverty and ill-health. Epidemics are likely to occur more frequently in the future, as heavy rainfall and flooding increase as a result of global climate change [1].

The World Health Organization (WHO) is currently working on an estimate of the global burden of human leptospirosis, through a review of the existing evidence, the development of new epidemiological tools to evaluate the burden of the disease, and identification of technical gaps for research [1]. It is estimated that there are more than 500,000 cases of leptospirosis worldwide, the majority of reported cases presenting severe manifestations with mortality greater than 10% [2].

Two species of the bacterium are recognized: *Leptospira interrogans *and *L. biflexa*. The species of importance for public health, *L. interrogans,* has more than 200 serologic variants, called serovars, grouped into 23 serogroups [3]. The serogroups are useful for understanding the epidemiological transition of the disease. There are universal serovars, such as *icterohaemorrhagiae* and *canicola*, as well as serovars that occur only in specific regions based on its ecology. Each serovar has a preferred animal host, but each animal species may act as a reservoir for one or more serovars [3].

The epidemiological cycle includes asymptomatic rodent carriers that act as maintenance hosts and infect other asymptomatic rodents, as well as wild animals, livestock, and domestic animals. After a week of leptospiremia, these animals shed leptospires in urine into their environment. Infection in humans usually occurs through direct contact with the urine of infected animals, or with a urine-contaminated environment such as soil, water, or plants. The bacterium enters the body through cuts or abrasions on the skin, or through the mucous membranes of the mouth, nose, and eyes [4]. Although more rare, studies have also shown direct transmission through lesions between humans and animals [3]. Wild and domesticated animals are essential for the maintenance of pathogenic leptospires in nature, while human-to-human transmission is unusual.

Difficulties in establishing a clinical diagnosis and the lack of diagnostic laboratory services are causes for underreporting in many countries. Clinical signs include fever, headache and myalgia, and may cause such complications such as jaundice, acute renal failure, bleeding including pulmonary haemmorhage syndrome, meningitis, myocarditis, and uveitis [1]. Cases are often variously misdiagnosed as meningitis, encephalitis or influenza, and outbreaks may be confused, or occur concurrently, with outbreaks of dengue or other viral hemorrhagic diseases, typhoid, rickettsial infection, malaria, or other febrile illnesses [5].

In Central America, one of the regions most vulnerable to natural disasters in the Americas, leptospirosis outbreaks have been reported in almost all countries [6,7,8,9,10]. Since 1995, leptospirosis outbreaks have been occurring frequently in Nicaragua. These outbreaks had been investigated, responded, and documented. Among them is the leptospirosis outbreak which occurred after hurricane “Mitch” in 1998, with 2,259 clinical cases documented in the literature [7,11,12]. From 2004 until 2010, several natural disasters have occurred in Nicaragua, most of which were water-related. Among them were two major events in 2007: the tropical cyclone “Felix”, a wind storm that affected close to 200,000 people on the Atlantic coast, and a flood which affected 24,000 people in the Northwestern part of the country. In 2010, another flood occurred in the same area. As a result of frequent natural disasters, the country has acquired significant experience in the control of the disease at the local level [7,11,12].

Floods or heavy rain are the possible drivers of leptospirosis outbreaks that have been most cited in publications of the region [7,12,13,14,15,16,17]. Other environmental factors such as the type of soil could also be possible drivers, since leptospira are known to survive longer in neutral to alkaline soil [3], alkaline surface water, and alkaline soil water [18]. Because it is a zoonotic disease, the presence of animals is also an important variable to explore. Another possible driver could be poverty and living conditions, as reflected in the occurrence of outbreaks in urban slum dwellers reported in various publications [19,20].

An analysis of the events recorded for WHO’s International Health Regulations (IHR 2005) in the American Region shows that outbreaks of leptospirosis were among the events of potential public health emergencies of international concern in the Americas in 2007 and 2008. The fact that 70% of these events were at the animal/human health interface highlights the need for a better understanding of infectious diseases common to man and animals [21]. Carrying out proper detection, risk assessment, and verification requires collaboration among the health sciences (veterinary and human) as well as other sectors. Leptospirosis is a disease that must be tackled within a multidisciplinary approach, both to understand its causality and to determine which actions must be taken against it. It is good example for the “One Health” approach [22,23].

This study was conducted for all the above reasons, in order to support countries in developing a holistic approach to prevention and response that brings together humans, animals and the environment. Its objective is to stratify the risk and identify “critical areas” for leptospirosis outbreaks in Nicaragua, and to perform an exploratory analysis of “drivers” for those outbreaks. The analysis of Nicaragua’s data will lead to the establishment of a methodology to identify possible risk areas for leptospirosis outbreaks in other Central American countries presenting similar drivers, and currently reporting few cases, as well as provide simple methodological steps to review existing surveillance and action plans related to this disease.

## 2. Methods

### 2.1. Type of Study, Period and Data Source

This is an ecological study at the second subnational level that includes the entire country (153 municipalities in 17 departments of Nicaragua) [24]. When it comes to describing outbreaks and interventions, it is retrospective and descriptive. The study’s time period is from 2004, when all departments started using ELISA tests for leptospirosis, to 2010, date of the latest available information. The data were obtained only through secondary sources. The number of human cases comes from the country’s information system, the human population, number of animals and socioeconomic variables from the 2005 Nicaragua Census (last information available when the study was performed), and the environmental data from other sources (list of variables in Table 1).

**Table 1:** Selected variables and sources of information used to create a database by municipality.

Variables taken from original sources	Sources
Name of the Department	Ministry of Health, Nicaragua [25]
Code of First National Sublevel	UN SALB [26]
Name of the Municipality	Ministry of Health, Nicaragua [25]
Code of Second National Sublevel	UN SALB [26] & Nicaragua Census [27]
Cases of Leptospirosis 2004–2010, by month	Ministry of Health, Nicaragua [25]
Population of Municipality 2005	Nicaragua Census [27]
Population of Municipality 2007	Ministry of Health, Nicaragua [25]
Rural Population in Municipality 2005	Nicaragua Census [27]
Area of Municipality in km^2^	UN SALB [26] & Nicaragua Census [27]
Percentage of People in Municipality Living in Poverty	Nicaragua Census [27]
Percentage of People in Municipality Living in Extreme Poverty	Nicaragua Census [27]
Population of 6-yr-olds and Over, Condition of Illiteracy in Municipality	Nicaragua Census [27]
Number of Bovine Animals in Municipality 2005	Nicaragua Census [27]
Number of Animals in Municipality, Pigs 2005	Nicaragua Census [27]
Number of Animals in Municipality, Horses 2005	Nicaragua Census [27]
	
**Variables created from original sources**	**Sources**
Percentage of Municipal Area Dedicated to Agricultural Land Use	FAO-GeoNetwork.a [28]
Percentage of Soil with Cambisol and Andosol Soil Type	FAO-GeoNetwork.b [29]
Percentage of municipal terrain with flat to moderate slope (25% or less)	USGS [30]
Minimum Precipitation in the Municipality per Year (mm)	Global Climate Data [31]
Maximum Precipitation in the Municipality per Year (mm)	Global Climate Data [31]
Average Precipitation in the Municipality per Year (mm)	Global Climate Data [31]
Variables calculated totals, rates and ratios	
Total number of cases (2004–2010)	
Cumulative incidence rate by 10,000 populations (2007)	
Percentage of illiteracy (2005)	
Bovine density (number of bovines/km^2^ (2005)	
Equine density (number of equines/km^2^ (2005)	
Swine density (number of swine/km^2^ (2005)	
Total animal density (number of animals/km^2^) (2005)	

### 2.2. Parts of the Study

The study was carried out in four stages that are described below.

#### 2.2.1. Collection of the Epidemiological History and Description of Actions Taken

This part consisted in a description of the major outbreaks that occurred in this period. The Nicaragua Leptospirosis Intersectoral National Plan was also reviewed, including the actions for prevention, alert and response for the control of leptospirosis in the country.

#### 2.2.2. Collection of Data and Analysis of the Epidemiological Situation, Including Risk Stratification and Identification of Critical Areas

The numbers of cases by municipality and department were collected and described by month and year, and a geo-coded municipal database was then created. An estimation of the cumulative incidence rate (per 10,000 populations) was calculated using the population of 2007. For the purposes of this study the following definitions were used:

1. Case definitions**: **Suspected case of leptospirosis—person with acute febrile illness with headache, nausea, vomiting, abdominal pain, arthralgia and prostration associated with conjunctival hyperemia, meningeal irritation, anuria or oliguria and/or proteinuria, jaundice and gastrointestinal and pulmonary tract bleeding. Probable case of leptospirosis—person with clinical signs and symptoms as well as history of exposure to pets or environment contaminated with animal urine or epidemiological link with a laboratory-confirmed case. Confirmed case of leptospirosis—Suspected persons with clinical symptoms of leptospirosis and laboratory confirmed diagnosis. For this database, only confirmed cases were included. The Ministry of Health of Nicaragua uses the same definition recommended by WHO that a case of leptospirosis requires laboratory confirmation [4].
2. Cumulative incidence, cumulative incidence rate (Syn: incidence proportion, average risk): The number or proportion of a group (cohort) of people who experience the onset of a health-related event during a specified time interval; this interval is generally the same for all members of the group, but, as in lifetime incidence, it may vary from person to person without reference to age [32].
3. Driver: Environmental or not individual socioeconomic factors that increase the probability of the event of an outbreak (increase of the incidence in a geographical area).
4. Hotspot: A geographical region where the concentration/cluster of the incidence of the disease is exceptional or in excess of the endemic rate, and where it can be predicted and will last for a period of at least a year [33,34,35]
5. Outbreak: An epidemic limited to localized increase in the incidence of a disease, e.g., in a village, town, or closed institution; upsurge is sometimes used as a euphemism for outbreak [32].
6. Risk factor: An aspect of personal behavior or lifestyle, an environmental exposure, or an inborn or inherited characteristic that, on the basis of scientific evidence, is known to be associated with meaningful health-related condition (s) [32].

For the purpose of this analysis, the criteria for risk stratification of leptospirosis in Nicaragua was defined using the following categories:

1. Productive area: area (in this case a municipality) where active transmission of leptospirosis cases is known to have occurred during the study period [36]. For this study, the productive area will be the addition of endemic and critical areas.
2. Silent area: area (in this case a municipality) where no cases were reported during the study period.
3. Endemic area: area (in this case a municipality) where active transmission of leptospirosis cases is known to have occurred in the period analyzed and that meets no criteria of critical area.
4. Critical area: area (in this case a municipality) where active transmission of leptospirosis cases is known to have occurred in the analyzed period and that meets at least one of the following two criteria: the municipality is in the top quintile of the cumulative incidence rate (10,000 populations) and/or the municipality is in the top quintile of the total number of cases. For the purpose of this part of the study to be used as evidence for decision makers to stratify risk of leptospirosis and appointed priority areas for intervention; it requires both criteria incidence rates and cases, because higher number of cases represents more resource allocation to respond to an outbreak such as medications, hospital beds, equipment and health personnel.

First, the risk stratification of the municipalities was done using an Excel spreadsheet to sort the criteria variables, and second, the geographic information system in epidemiology (SIGEpi) developed by PAHO was used to select the top quintiles of these two indicators using the tool “Identification of Priority Areas”, in order to determine the critical areas on the maps and complete related tables.

#### 2.2.3. Collection and Processing of Environmental, Demographic and Socioeconomic Variables

Nicaragua’s digital cartographic data were collected, updated, standardized and geo-processed using ArcGIS/Editor 10.0. The first step was to update the municipal boundaries available in the UN SALB Project [26] following the boundary depiction released by the Nicaraguan National Institute of Statistics. Area-specific statistics of precipitation patterns and slope were then calculated using ArcGIS/Editor/Zonal Statistics. The variables created to analyze precipitation in this study were calculated from open access source with a very high resolution and robust series of 50 years of precipitation, which we measured by municipality extracting mean, median, minimum, maximum and other monthly statistics. In climatology, this means the best possible scenario to avoid biases and annual variations and to identify a regular rain pattern.

The occupied municipal surface was then calculated with dominant soils, agricultural-livestock land use and flat-moderate slope using ArcGIS/Editor/Extract by Mask, converting raster to feature, calculating geometry, and using other Geo-processing techniques-intersection & dissolving- to get the municipal area statistics. All demographic and socioeconomic data were captured from Nicaragua’s Census and included in the geo-coded database in DBS-Excel. The construction of rates and ratios yielded new variables (Table 1).

#### 2.2.4. Exploratory Geo-Referenced and Statistical Analysis of Possible Drivers Using Selected Environmental, Demographic and Socioeconomic Variables

Since one of the objectives of this study is to explore the possible drivers to leptospirosis outbreaks, different methodologies of geo-processing information and statistics analysis were used in this subsection of the study.

The 16 variables selected as potential environmental, demographic and socioeconomic drivers for this study were analyzed geographically, using choroplet map overlapping with critical areas of leptospirosis defined by the top quintiles of incidence rates only. Thematic maps were produced, also using quintiles, to explore the possible associations geographically with the critical areas. Only those relevant maps were presented in the results.

Exploratory statistical analysis to measure possible association of these 16 variables, with critical areas of leptospirosis defined only by the top quintile of incidence rate, were developed with the following steps:

1. Univariate analysis, reporting the mean and standard deviation. Followed by a bivariate analysis, Pearson correlation with the cumulative incidence rate (per 10,000 populations) were calculated for each selected variable using SAS 9.2.
2. A dichotomous variable was created using Microsoft Excel 2010 which identifies each municipality considered as a critical or a non-critical area based on whether or not the municipality is in the top quintile of the incidence rate.
3. A simple univariate cluster procedure for the incidence was used based on the criteria of minimization for within-class variability.
4. Critical and non-critical municipalities, as well as classes of municipalities yielded by the univariate cluster analysis were compared for exploratory purposes. Coincident statistically significant variables in both analyses were used to fit various regression models as described below.
5. The comparisons were done as part of the initial exploratory analyses by means of *t*-test procedures and one-way ANOVA, and bear no inferential purposes for causal relationships whatsoever.
6. A binary logistic regression model was fit in which a dichotomous variable identifying critical and non-critical municipalities was the dependent variable, and the predictors yielded by the previous exploratory analysis were the explanatory variables.
7. A multinomial logistic regression analysis was also fit with the nominal variable that came out of the univariate cluster analysis which was used as the dependent variables.
8. Finally, a Poisson regression model was performed using the same explanatory variables with the incidence rates as dependent variable.
9. A lagged cross-correlation analysis was performed to determine the association between rain pattern and case distribution by month. SAS 9.2 was used to perform for this analysis.

## 3. Results and Discussion

### 3.1. Description of the Situation

Nicaragua, is divided into 17 departments (at the first subnational level). The country has a population of 5,142,098 according to the 2005 Census, where 54.38% reside in the Pacific Region, 33.47% in the Central Region, and 12.15% in the Atlantic Region. The departments of the Pacific Region have a higher population density and probably have easier access to health units. The Atlantic Region, however, has a higher percentage of its population residing in rural areas. In the densely populated Pacific Region, the natural ecosystems have been altered by economic activities; whereas the Atlantic Region preserves more of its natural environment with its sparsely distributed communities. During the rainy season, transportation routes leading to health care units in remote areas may become more difficult. For these remotes areas, case detection and laboratory confirmation may be more limited.

Agriculture is important for the economy and the everyday lives of the population for this country which spans an area of 130,000 km^2^. The livestock with the highest number in Nicaragua was bovine, with was a total number of 2,657,039 in 2005. The number of equine stock was 383,172, and swine was 412,604 contributing to a total of 3,452,815 animals. The average ratio of livestock by population within the municipalities was 1.02 (range: 0.01–6.25), and this ratio suggested that there are areas with a higher number of animals than people. The main crops produced in Nicaragua are rice, corn, sorghum, peanuts and coffee.

In total, there are 153 municipalities (at the second sub-national level) which vary in size from coast to coast. In the Pacific Region (Western), the municipalities are usually smaller in area, while the municipalities of the Atlantic Region (Eastern) are larger in area and less populated.

Each department belongs to the local health system (Local System of Integrated Assistance in Health of the Ministry of Health; SILAIS for its Spanish acronym). The system provides free medical care to the population. In Nicaragua, leptospirosis prevention and control activities currently take place in the 17 SILAIS. The country has an Intersectoral National Plan of Leptospirosis which was developed by the Ministry of Health, Ministry of Agriculture and Forestry, Leon University with the support Pan American Health Organization (PAHO).

Until 2000–2002, the diagnosis of leptospirosis was based on clinical management and laboratory confirmation through micro agglutination test (MAT), a technique only available at Nicaragua’s National Center for Diagnosis and Reference (CNDR from its Spanish acronym). As of 2003, active surveillance of cases with fever was initiated, using a standardized ELISA test available in laboratories of the national network present in all SILAIS. In 2008 and 2009, active surveillance was put in place to detect cases using the techniques mentioned above. Since 2010, besides using ELISA as a screening method, a rapid test is performed at local level, which enhances early detection and the management of outbreaks at primary care level.

Samples are sent to the Central Laboratory to confirm the diagnosis using MAT which is considered the gold standard by WHO [4]. The ELISA IgM test, used for diagnosing leptospirosis in human samples, is reported to have 100% sensitivity and **≥**99.6% of specificity in comparison to the MAT. ELISA IgG and serology tests are also performed on animal samples in different species by the Central Laboratory and also by Leon University. In many cases, the serovar is identified from the human and animal samples. More than 50 serovars were identified in country, and, among them are *serjroe*, *canicola*, *australis*, *louisiana*, *icterohaemorrhagie *and *bataviae*.

In the period of this study, between 2004 and 2010, several outbreaks were reported in Nicaragua. Outbreak reports indicate that most of them were in León and Chinandega, in 2007 (338 cases), 2008 (146 cases), and 2010 (248 cases). Several cases presented co-infection with dengue. Reports from the outbreak investigation show that in 2007 and 2010 in León, around 77% of the cases came from rural areas, and 75% were males. In the Chinandega outbreak, around 50% of the cases were among people between 15 and 49 years of age. The outbreak data disaggregated by individuals suggest that the risk factors are related to occupation (males of reproductive age living in rural areas).

The actions put in place in both departments to control the outbreak started with the creation of multidisciplinary municipal teams. Antibiotic treatment was administered to the human cases, and chemoprophylaxis was used for their contacts. On the animal side, samples from different animals were analyzed to investigate the possible sources with positive samples in different species. Activities were carried out to detect and treat leptospirosis in domestic animals. Animal vaccinations were done by their owners with commercial vaccines available. The response also included the control of rodents, community participation, training for animal and human health workers, and health communication. An ongoing, unpublished study is being developed currently by Leon University analysing seroprevalence on rodents polulation. In the Department of Leon, the rodents captured in highest number were *Mus musclulus *followed by *Ratus ratus*. A considerable percentage of these animals have been confirmed positive for leptospirosis.

### 3.2. Epidemiological Description and Risk Stratification

Between 2004 and 2010, 1,980 cases of leptospirosis were reported in Nicaragua. The number of cases by year ranged between 56 (2004 and 2006) and 685 (2007), with a high number of cases (653) in 2010 as well. León was the department with the highest percentage (26.36%), followed by Chinandega (15.35%) and the most populated department, Managua (8.84%), where the national capital city is located. Together those three departments present 50.55% of the total of cases in the period (Table 2). The number of deaths was: zero in 2004, 2005 and 2006; twelve in 2007; five in 2008; one in 2009 and twenty in 2010. The fatality rate for the period was 1.41%.

**Table 2:** Cases of leptospirosis by department, percentage of cases and rates by 10,000 populations, Nicaragua, 2004–2010.

	Cases								% of total cases	Rate (10,000)
Dept.	2004	2005	2006	2007	2008	2009	2010	Total		
Boaco	4	6	0	33	13	3	31	90	4.55%	4.48
Carazo	10	2	1	2	2	1	95	113	5.71%	7.35
Chinandega	4	7	7	121	99	7	59	304	15.35%	18.84
Chontales	1	0	1	36	2	13	24	77	3.89%	6.73
Estelí	0	0	0	19	35	0	39	93	4.70%	5.77
Granada	1	1	1	15	6	1	12	37	1.87%	2.21
Jinotega	11	3	5	28	8	5	29	89	4.50%	13.93
León	2	44	6	229	48	7	186	522	26.36%	36.03
Madriz	0	2	2	4	14	3	8	33	1.67%	3.67
Managua	0	0	2	30	44	22	77	175	8.84%	7.60
Masaya	1	7	7	3	3	5	8	34	1.72%	3.94
Matagalpa	2	0	3	84	16	22	24	151	7.63%	6.51
Nueva Segovia	8	1	12	19	12	7	11	70	3.54%	6.93
R.A. Atlántico Norte	2	1	0	15	2	5	6	31	1.57%	0.95
R.A. Atlántico Sur	3	3	7	22	6	18	22	81	4.09%	4.13
Río San Juan	6	1	2	22	13	5	10	59	2.98%	6.90
Rivas	1	4	0	3	1	0	12	21	1.06%	1.60
Total	56	82	56	685	324	124	653	1,980	100%	6.51 (median)

Source: Ministry of Health of Nicaragua [25]. Analysis was carried out by the authors.

**Figure 1:**
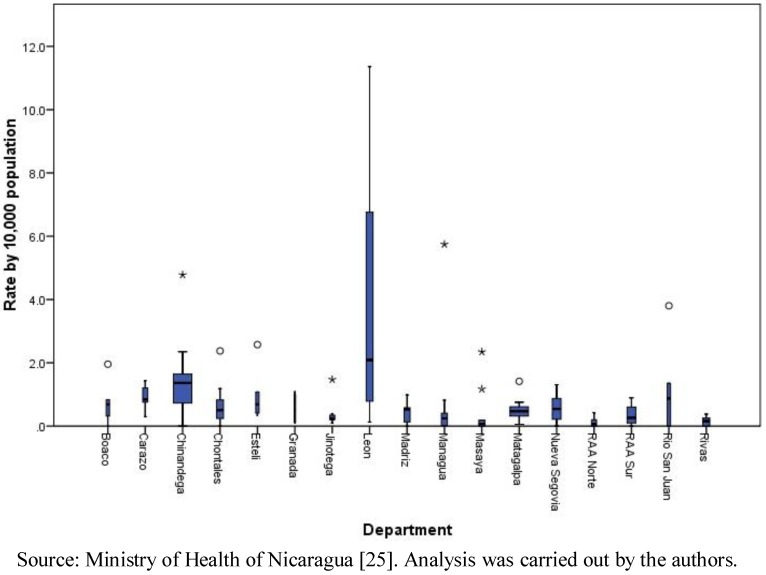
Leptospirosis municipal rates by 10,000 populations, by department, Nicaragua, 2004–2010.

**Figure 2:**
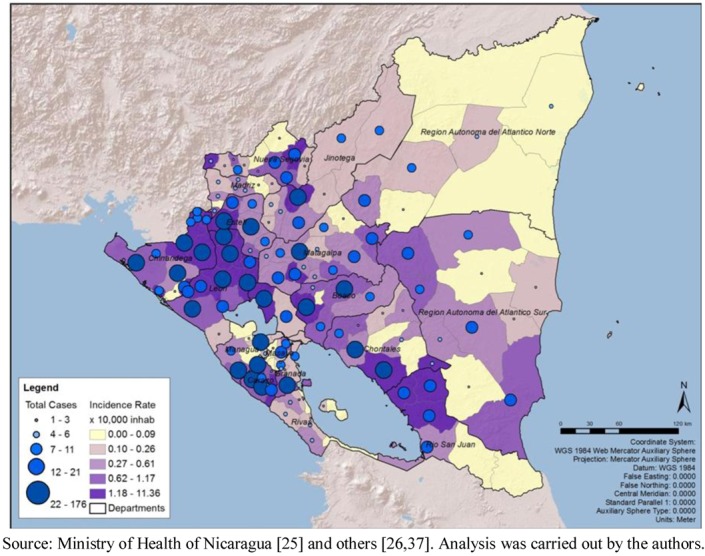
Total number of cases of leptospirosis, cumulative incidence rate (10,000 populations), by municipality, Nicaragua, 2004–2010.

At the municipalities level, the range of the total number of cases was 0 to 177, with a median of six cases and a mean of 12.94 (standard deviation of 21.98). The total number of cases by month in the period ranged from 26 in April to 1,081 in October. The cumulative incidence rate (per 10,000 populations) by department confirms the result mentioned above that León (with a rate of 36.03 per 10,000 populations) and Chinandega (18.84) presented the highest rates in the country. The municipal rates ranged between 0 and 11.36, with a median of 0.41 and a mean of 0.83 (standard deviation of 1.45). Clearly, León presented the highest municipal rates among all (Figure 1). The geographic distribution of total cases and cumulative incidence rates showed a concentration of highs in the municipalities of the Pacific Region (Figure 2).

**Figure 3:**
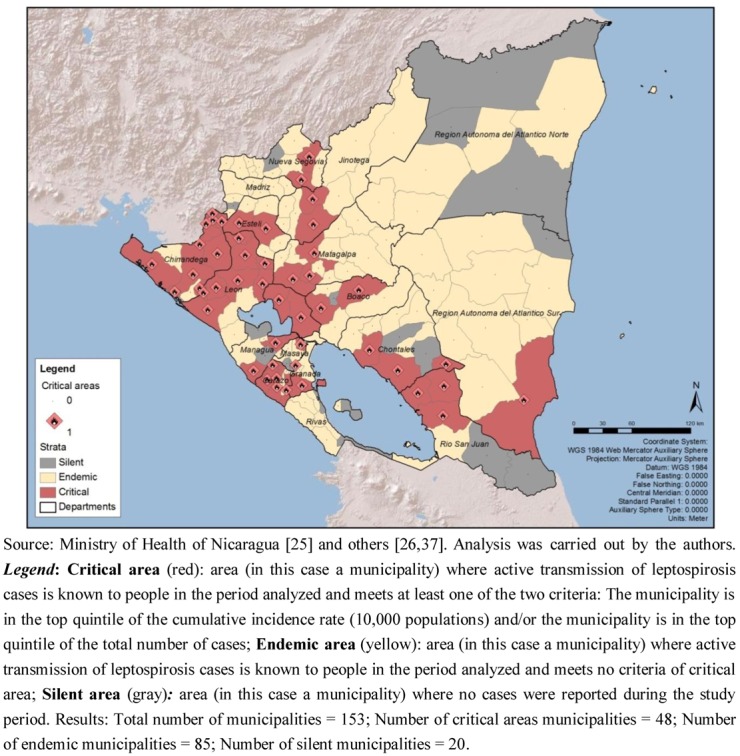
Risk stratification of leptospirosis in Nicaragua, by municipality, 2004–2010.

The analysis of the risk stratification criteria established for this study showed that out of the 153 municipalities, 133 were productive for leptospirosis:

1. 48 were critical area municipalities (31.37% of the total number of municipalities). 31 were considered critical areas since the municipality is in the top quintile of the cumulative incidence rate (10,000 populations); and 32 since the municipality is in the top quintile of the total number of cases; 15 of the municipalities overlap both criteria.
2. 85 were endemic municipalities (55.56%).
3. 20 were silent municipalities (13.07%) (Figure 3).

The departments with 50% or more critical area municipalities were León (80.00%), Chinandega (76.92%), Carazo (62.50%) and Rio San Juan (50.00%). Most of the critical areas were located on the Pacific coast. On the other hand, the departments that presented 30% or more municipalities considered as silent areas were Región Atlántica Norte (37.50%), Rio San Juan (33.33%), Masaya (33.33%) and Rivas (30.00%) (Table 3).

**Table 3:** Population, number of municipalities, risk stratification, by department, Nicaragua, 2004–2010.

Department	Population	Number of municipalities	Municipalities					
			Critical areas%		Endemic%		Silent%	
Boaco	155,155	6	2	33%	3	50%	1	17%
Carazo	171,055	8	5	63%	3	38%	0	0%
Chinandega	390,339	13	10	77%	2	15%	1	8%
Chontales	158,550	10	3	30%	5	50%	2	20%
Estelí	207,603	6	2	33%	4	67%	0	0%
Granada	173,204	4	1	25%	3	75%	0	0%
Jinotega	306,795	8	2	25%	6	75%	0	0%
León	365,539	10	8	80%	2	20%	0	0%
Madriz	136,433	9	0	0%	8	89%	1	11%
Managua	1,300,867	9	4	44%	3	33%	2	22%
Masaya	298,688	9	2	22%	4	44%	3	33%
Matagalpa	483,247	13	3	23%	10	77%	0	0%
Nueva Segovia	214,779	12	2	17%	9	75%	1	8%
R. A. Atlántico Norte	323,554	8	0	0%	5	63%	3	38%
R. A. Atlántico Sur	315,705	12	1	8%	10	83%	1	8%
Río San Juan	98,464	6	3	50%	1	17%	2	33%
Rivas	160,971	10	0	0%	7	70%	3	30%
Total	5,260,948	153	48	31%	85	56%	20	13%

Source: Ministry of Health of Nicaragua [25,27]. Analysis was carried out by the authors.

### 3.3. Exploratory Analysis of Possible Drivers

**Figure 4:**
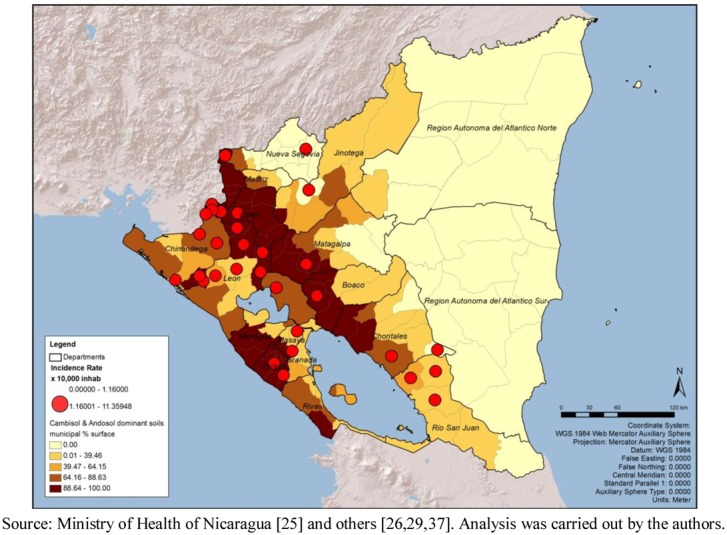
Critical areas for leptospirosis define by incidence rate and percentage of soil with Cambisol and Andosol, by municipality, Nicaragua, 2004–2010.

The municipal mean, standard deviation, and Pearson correlation of possible drivers with the cumulative incidence rate are presented in Table 4. The variable presenting the most visible pattern of spatial overlap with critical areas due to incidence rate is the percentage of municipal surface occupied by the soil combination of cambisol and andosol (Figure 4). The Pearson correlation of this variable with the incidence rate showed a positive strength (r = 0.275; *p* < 0.01). Cambisol is young, less-structured soils with diverse parental material [38], however in this case they are settled over Ignimbrite-pyroclastic bedrock and lava strata of andesite and basalt. Andosol is located over a volcanic ash foundation [38,39]. This analysis was carried out ex-post-facto based on direct communication with local Nicaraguan biologists and engineers about the role of soils in leptospirosis distribution.

**Table 4:** Municipality mean, standard deviation, and correlation among selected possible drivers and cumulative incidence rate (10,000 populations), Nicaragua.

Possible drivers	Municipality mean	Standard deviation	Correlation with cumulative incidence rate
% rural population	61.317	24.246	0.207 **
			*p* = 0.0104 *
Population density	1.350	3.215	–0.107
			*p *= 0.188
% illiterate population	24.744	9.543	–0.015
			*p * = 0.8538
% population living in poverty	30.366	5.469	0.150 **
			*p * = 0.0647
% population living in extreme poverty (n = 151)	43.191	16.727	–0.004 **
			*p * = 0.9641
Minimum rain precipitation per year (mm)	165.660	204.701	–0.239 **
			*p * = 0.0029 **
Maximum rain precipitation per year (mm)	3,250	887.127	0.030 **
			*p * = 0.7147
Average rain precipitation per year (mm)	1,459	536.998	–0.147 **
			*p * = 0.0696
Average rain precipitation of the two months with the highest rainfall during the year (mm)	7,403	1,490	0.181 **
			*p * = 0.0254 *
Bovine density	27.384	1.780	0.077 **
			*p * = 0.343
Equine density	4.6842	2.461	0.075 **
			*p * = 0.356
Swine density	5.061	4.846	–0.052 **
			*p * = 0.524
Animal density	37.130	2.100	0.062 **
			*p * = 0.444
% land area dedicated to agricultural land use	66.016	32.928	0.097 **
			*p * = 0.2351
% soil type with cambisol and andosol	51.794	42.470	0.275 **
			*p * = 0.0006 **
% municipal terrain with flat to moderate slope (25% or less)	41.591	28.631	0.0002 **
			*p * = 0.9977

* significant *p* < 0.05, ** significant *p* < 0.01.

**Figure 5:**
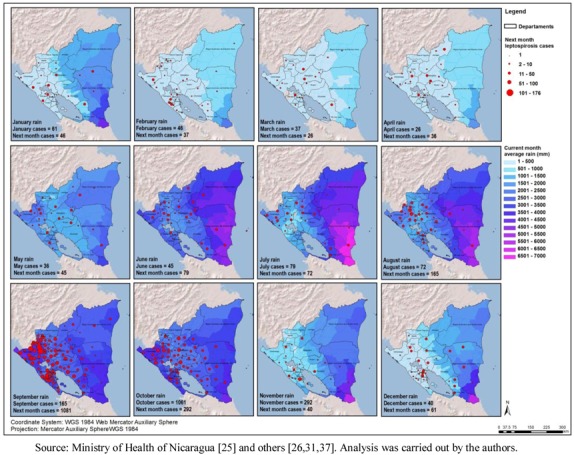
Average rainfall and total number of cases of leptospirosis, in the next month, by municipality Nicaragua, 2004–2010.

Another environmental variable explored in this study was precipitation or rainfall. The minimal precipitation per year (mm) variable was negatively correlated with the leptospirosis distribution (r = 0.239; *p* < 0.01). The average pluvial precipitation of the two months with highest rainfall (mm) also presented a positive correlation (r = 0.181; *p* < 0.05). However, the most informative set of maps was the monthly average precipitation analyzed together with the number of cases in the same month and in the following month (Figure 5). The months of March and April showed the lowest precipitation, and the average monthly numbers of cases were 37 and 26, respectively, during the time period of the study.

**Figure 6:**
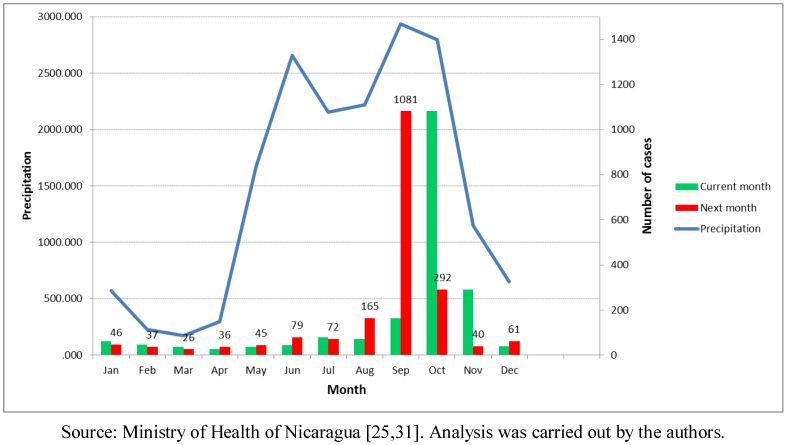
Annual rain pattern and leptospirosis cases distribution in the current and next month, Nicaragua, 2004–2010.

The months of September and October were the ones with the highest precipitation in the Pacific Region, and also the highest average number of leptospirosis cases. From 185 average total cases in September, the number jumped to 1,081 cases in October in the period. The Atlantic Region, has a different rain pattern where higher precipitation is common throughout most of the year. Furthermore, it has a more natural environment in comparison to the Pacific Coast. For the entire country, the rain started increasing in June followed by a second rise in September, The number of cases maximizes after the precipitation is highest in the month of September (Figure 6). Additional analysis showed a positive time lag association between amount of rain and number of leptospirosis cases. The cross-correlation analysis performed between annual rain patterns and case distribution by month showed a higher correlation with the previous month rainfall (0.558) than with the concurrent month (0.441).

**Figure 7:**
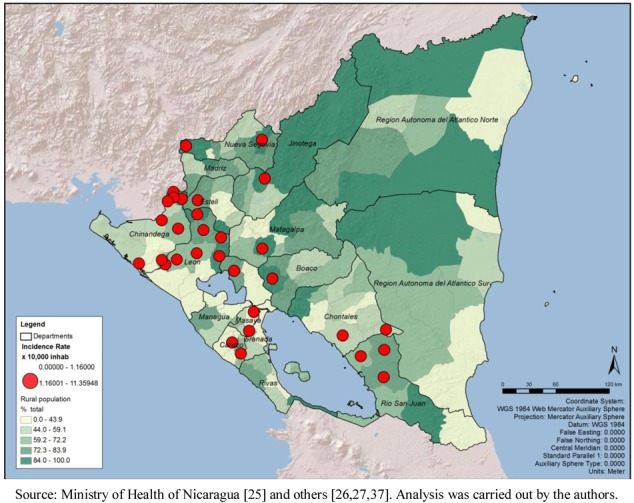
Critical areas for leptospirosis define by incidence rate and percentage of rural population, Nicaragua, by municipality 2004–2010.

In the last Census available during the study (2005), Nicaragua has a population of 5,142,098, 61.32% of which is rural; the percentage of rural population presented a positive correlation with the incidence rate (r = 0.207; *p* < 0.01). Visualizing the map of percentage of rural population distribution it does not present a clear association with the critical areas; this could be the result of the municipalities being larger and more rural in the Atlantic Region (Figure 7). Among the 31 municipalities in the top quintile of incidence rates, 18 of them have 72% or more of rural population. 

**Figure 8:**
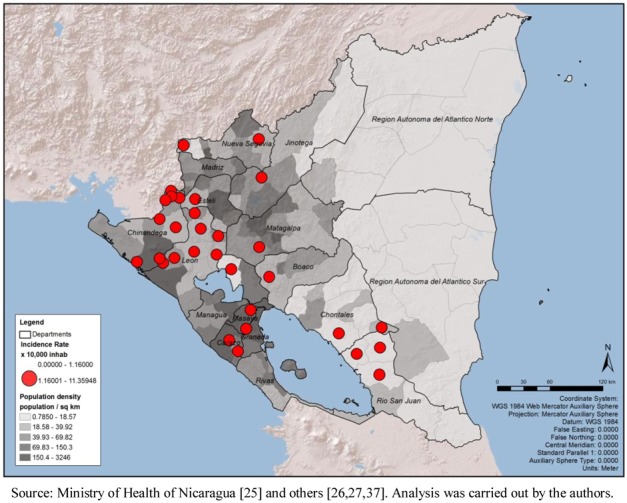
Critical areas for leptospirosis define by incidence rate and population density, Nicaragua, by municipality 2004–2010.

The population density (ratio of population/km^2^) shows a higher concentration of people living in the Pacific Region, surrounding the capital area of Managua as well as major urban areas of Chinandega and Leon; no clear association with the critical areas for leptospirosis outbreaks could be suggested (Figure 8). Even though this variable does not suggest association with the critical areas, by spatial or statistical correlation, the map was retained in order to show that population density is probably not a bias in this analysis.

The percentage of population living in poverty (mean 30.36%, range: 11.50–43.30%) was among the socioeconomic variables analyzed as possible drivers, which in the case of Nicaragua is measured as having one type of basic unmet needs. In the case of Nicaragua, extreme poverty (mean 43.19%, range: 4.30–87.40%) is measured as two or more basic unmet need. Along with illiteracy (mean 24.74%, range: 7.91–56.23%), it is highest in the Atlantic coast; however the environmental conditions are different in both coasts.

A possible driver that was also analyzed was the percentage of area dedicated to agriculture land use and the density of animals per km^2^ (Table 4). No association was found with the percentage of area dedicated to agricultural and livestock land use. Another set of variables were created for animal density per km^2^ (number of bovine, equine, swine and their total per km^2^) were analyzed; however, no association with the critical areas was suggested using the data aggregated by municipality.

As part of the exploratory analysis of this subsection using several methodologies, Table 5 presents the municipality mean, standard deviation, and *t *test among the selected possible drivers and the dichotomous variable, critical area and non-critical area, due to incidence rate (represented by 1 and 0, respectively). For some variables the differences between the means of critical area and non-critical area are large, such as in the case of population density (critical = 69.82; non-critical = 151.62 population per km^2^) and minimal rain precipitation per year (critical = 69.24; non-critical = 190.92 mm). The results in this table strengthen the observation made in the previous analysis using the thematic maps and the Pearson correlation.

**Table 5:** Critical (1) and non-critical (0) areas define by incidence rate, number of municipalities, mean, standard deviation, and *t *test among selected possible drivers, Nicaragua.

Incidence rate (dichotomous variable)	N		Mean	Standard deviation	*t* test for equality of means *	
					*t*	Sig. (2 tailed)
% rural population	1	31	72.54	18.62	2.958	0.004
	0	122	58.47	24.74		
Population density	1	31	69.82	97.81	–1.267	0.207
	0	122	151.62	355.17		
% illiterate population	1	31	24.70	6.48	–0.027	0.979
	0	122	24.75	10.20		
% population living in poverty	1	31	32.20	3.63	2.111	0.036
	0	122	29.90	5.76		
% population living in extreme poverty (n = 151)	1	31	42.51	10.91	–0.041	0.968
	0	122	42.65	18.65		
Minimum rain precipitation per year (mm)	1	31	66.24	80.74	–3.114	0.002
	0	122	190.92	218.74		
Maximum rain precipitation per year (mm)	1	31	3,315.97	629.39	0.459	0.647
	0	122	3,233.77	942.88		
Average rain precipitation per year (mm)	1	31	1,324.54	280.49	–1.571	0.118
	0	122	1,493.40	580.44		
Average rain precipitation of the two months with the highest rainfall during the year (mm)	1	31	8,000.63	1,524.31	2.544	0.012
	0	122	7,251.60	1,448.56		
Bovine density	1	31	30.82	17.69	1.203	0.231
	0	122	26.51	17.80		
Equine density	1	31	5.28	1.96	1.520	0.131
	0	122	4.53	2.56		
Swine density	1	31	4.88	3.26	–0.235	0.814
	0	122	5.11	5.18		
Animal density	1	31	40.97	20.48	1.143	0.255
	0	122	36.15	21.10		
% land area dedicated to agricultural land use	1	31	72.69	29.86	1.267	0.207
	0	122	64.32	33.57		
% soil type with cambisol and andosol	1	31	70.45	38.68	2.800	0.006
	0	122	47.05	42.23		
% municipal terrain with flat to moderate slope (25% or less)	1	31	45.09	33.13	0.762	0.447
	0	122	40.70	27.45		

* Equal variances assumed.

Three clear-cut classes were identified by the univariate cluster analysis. There is a remarkable difference between the high rates class and the other two classes. Very high rates are typically concentrated in a few municipalities in Nicaragua (Table 6).

**Table 6:** Average incidence rates (centroids) and typical incidence rates as results of a univariate cluster analysis.

Classes	N	Centroids	Typical rate
Low rates	122	0.35	0.34 (a)
Medium rates	26	1.75	1.64 (b)
High rates	5	7.48	6.76 (c)

(a) Santo Domingo, Chontales; (b) San Fco del Norte, Chinandega; (c) El Jicaral, León.

The comparisons were done as part of the initial exploratory analyses by means of *t*-test procedures and one-way ANOVA, and bear no inferential purposes for causal relationships whatsoever. The same four variables were identified by both analyses: minimum precipitation, percentage of Cambisol and Andosol soil type, percentage of rural population, and average rainfall in the two months with more precipitation (Table 7).

**Table 7:** Comparisons of the different exploratory analysis.

Variable	Model							
	Binary logistic		Multinomial logistic (a)		Multinomial logistic (b)		Poisson	
	Wald test	*p* value	Wald test	*p* value	Wald test	*p* value	Wald test	*p* value
% rural population	2.14	0.144	12.74	0.000	4.37	0.037	739.1	0.000
% soil type with cambisol and andosol	0.32	0.574	1.15	0.284	0.31	0.580	46.1	0.000
Minimum monthly rain	3.90	0.048	9.95	0.002	2.57	0.109	223.4	0.000
Average rain precipitation of the two months with the highest rainfall during the year (mm)	5.42	0.20	6.94	0.008	0.62	0.430	190.4	0.000

(a) Class 2 *vs*. class 1; (b) Class 3 *vs*. class 1.

Precipitation emerges as a relevant explanatory variable in all models except for the multinomial logistic model (b). This multinomial model takes class 1 as a reference and identifies relevant factors in the other two classes. The very high incidence class is separated from the medium incidence class due to a higher percentage of rural population. All variables are highly significant to predict incidence rates. However, the best model that could be suggested for this exploratory analysis is a Poisson regression using incidence rate as a continuous variable.

### 3.5. Limitations of This Ecological Study

Commonly associated with this type of study is the ecological fallacy [32]. However, the purpose of this study is to support interventions in the countries that are usually divided by administrative areas and also to explore possible drivers using available information. In spite of its apparent limitations, this ecological study is advantageous since it was developed quickly and inexpensively using existing epidemiological and census data from the country’s information system and open access environmental databases from the international agencies’ web pages. This ecological municipal approach would probably be more easily reproduced and feasible for countries with similar challenging conditions. Since strong associations are detected in this study, further analysis could be developed using others disaggregated designs. Individual factors such as behavior related to the use and proximity of bodies of water and animals could not be measured in this design.

Similarly, there is a very fine line separating visualization and exploratory spatial data since both of them share graphical methods. Nevertheless, as we analyzed the distribution of health problems applying statistical classification methods to categorize the municipal units; zonal statistics to identify rain patterns; and geo-processing techniques to measure the environmental factors distributed inside the administrative areas, we therefore refer to this study as an exploratory spatial study.

Other variables were analyzed and mapped, but they did not show a clear statistical association, or strong geographic correlation patterns at this level of analysis. An additional set of considerations may be related with the differences between the East and West coasts of Nicaragua, as the environmental, economic and social factors vary substantially. Characterizing the dynamics of leptospirosis transmission in each region could be a future step. The natural dynamics seem to be clear for the Pacific Region: a concentrated and bimodal rain pattern with potential flash floods, soil and bedrock with igneous origin, altered ecosystems, and more intensive land use. In this study, our goal was to analyze the entire country, and, since we have managed to characterize the difference in patterns between the two coasts, in the future, studying the East and West coasts separately could be a good methodological approach. 

## 4. Conclusions

This study demonstrated that there are different risk areas for leptospirosis in Nicaragua. Most of the critical areas that were identified through the present methodology are concentrated in the Pacific region, confirming previous descriptions cited by the national authorities in the Intersectorial National Leptospirosis Plan for Nicaragua [40]. Three of the 17 departments (León, Chinandega and Managua) presented half of the human cases detected in the country during the period under study. León and Chinandega have the highest risk as measured by the number of cases, the highest cumulative incidence rate, and also the highest percentage of municipalities considered as critical areas. These findings also confirmed previous publications about outbreaks in 1995 and 1998 in these areas [7,11,12]. These departments and most of their municipalities need to remain a priority for intervention in order to avoid large outbreaks.

The highest percentage of municipalities (56%) was classified as endemic areas, where the disease is present but not within the highest quintiles. Thirteen percent of the municipalities in Nicaragua had no case of leptospirosis, which could suggest difficulties in the surveillance even though the ELISA test is implemented in all Departments. These municipalities are mostly in remote areas in the Atlantic region. To analyze if leptospirosis is really absent in silent areas and to confirm the burden of the disease in the other areas, we suggest creating an indicator of reliability for the surveillance. One option could be to use the proportion of rapid tests for leptospirosis related to the number of reported cases with fever. A similar type of indicator (annual proportion of dog samples for rabies test related to the dog population) has been used in Brazil for almost 20 years, and was later extended to Latin America [36,41].

In this study, a stratification of risk is proposed based on two criteria to define critical areas both related to magnitude (number of cases and cumulative incidence rate). The frequency of events (outbreaks) is another variable that could be analyzed in the future and that may be added to this methodology when it is applied to another unit of analysis.

The fatality rate for leptospirosis has been reported to range from 0.5% to 30% worldwide [4]. According to the WHO, the majority of the reported cases for leptospirosis have severe manifestations, for which the mortality is greater than 10% [1]. The fatality rate for Nicaragua analyzed in this study was 1.41%, this percentage is on the lower range of reported studies. This could suggest that the country is responding quickly to outbreaks, and facilitating early detection and management of severe cases at primary healthcare level.

During the period under study, the description from outbreaks revealed that many leptospirosis cases were co-infected with dengue. This information is very important, as it suggests that training of health personnel in the adequate diagnostic test for the disease is crucial to avoid severe cases and mortality. This also implies that laboratory diagnosis of leptospirosis is available.

**Figure 9:**
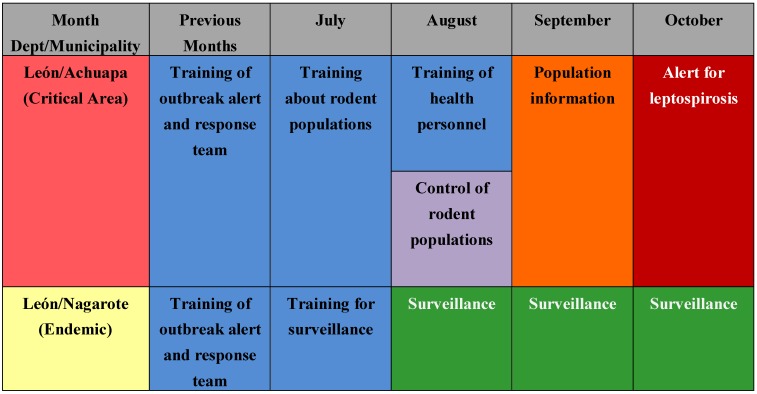
Example of possible use of this information for planning to prevent and respond to leptospirosis outbreaks in the case of Nicaragua.

The association of leptospirospis with rain is mentioned in several publications and measured in a few others [14,15,42,43,44], and our study confirms this association. Figure 5 shows the monthly precipitation and the number of cases in the following month. The number of cases increased more than ten times during the rainy season. For countries such as Nicaragua with a clear rainy season (around October in the Pacific region) and where critical areas have been identified, this information could support the planning of monthly prevention and response activities for leptospirosis outbreaks, such as training of personnel in different aspects, alert about the disease, and others. For other areas not considered as critical areas, surveillance and readiness may be sufficient (example in Figure 9). It is crucial that a leptospirosis plan take into consideration all the components of a multidisciplinary team and partnerships with others sectors, such as health care, surveillance/diagnosis, action in livestock, rodent control, communication, and others. An interprogramatic collaboration with teams working on other issues such as dengue, influenza and natural disasters is also necessary.

The analysis of possible drivers for leptospirosis outbreaks is exploratory. The variable that shows a clear relation with critical areas defined only by incidence rates was the percentage of cambisol and andosol as dominant soils. More than half of Nicaraguan territory has a geologic igneous origin [39]. Several volcanoes, not all active, are located along the Pacific coast, where most of the critical areas are found. This physical condition may facilitate longer survival of the bacteria in the soil/water and will not change in time. According to Gordon [18], “the incidence of leptospirosis is dependent on the survival time of shed leptospires in surface water or soil water” which in turn is affected by the water acidity-alkalinity level. This, added to the humidity levels generated by substantial rainfall occurring during part of the year in that area, suggests that the country needs to be prepared for other leptospirosis outbreaks.

The results of the exploratory analyses of possible environmental, demographic and socioeconomic drivers using different methodologies, including GIS and four statistical models identify four variables as the most important: percentage of Cambisol and Andosol soil type, minimum precipitation, average rainfall in the two months with more precipitation, and percentage of rural population.

According to the Center for International Earth Science Information Network (CIESIN), Nicaragua is one of the top 15 countries exposed to three or more natural hazards (measure based on affected land area), with a total exposed territory of 3.0%, and 22.2% of its population exposed as well. Among the hydro-meteorological hazards that were analyzed, extreme flood events and tropical cyclones affect vast areas of the entire Nicaraguan territory; comparatively the geophysical hazards such as earthquakes (analyzed through their probability of occurrence, strength greater that 4.5 on the Richter scale, and volcanic activity, and measured according to their explosivity) affect less extensive areas on the west coast [45,46].

In other Central American countries, similar environmental conditions such as dominant soils, rain patterns and igneous-volcanic geologic origin and ecosystem conditions are probably present. There are 80 volcanoes in the sub region along the isthmus’s Pacific edge, located along the Ring of Fire [46,47]. This combination of factors suggests that the problem may be broadly spread in the area.

These conditions demonstrate the importance of international health cooperation, including financing, to assist the country in dealing with these physical threats and their indirect consequences, such as an increase in the number of communicable diseases that could affect Nicaragua. The Nicaraguan population already has one of the lowest Gross National Income (purchasing power parity value) of the Region [48].

However, Nicaragua has the most number of reported cases. Strengthening the surveillance of leptospirosis will perhaps yield different results regarding the burden of this disease. As expressed by Aron [49], “a better understanding of the dynamic linkage between ecosystems and public health is leading to a new diverse opportunity for interventions earlier in process that could become direct threats to public health”.

Outbreaks with a large number of cases do not occur every year. The years with most cases during the study period were 2007 and 2010. These two years also coincided with the occurrence of natural disasters [49,50]. Most tropical storms reached Nicaragua through the Atlantic coast where other environmental conditions are not as prominent, and which are also less populated. The fluctuations in precipitation levels may be enough to increase the number of cases. Further analysis including other environmental variables related to flood, ecosystems, and soil are necessary for a deeper understanding of possible environmental drivers for leptospirosis.

We also suggest future studies focusing on livestock at a more disaggregated level because this will open the possibility of, not only analyzing the presence of animals in critical areas, but also studying what types of serovars are circulating in these areas. In Nicaragua, there is one bovine per two people, which suggests the economic importance of cattle ranching to the country. The type of productive process in large or small livestock and whether it raises the risk of leptospirosis could also be analyzed.

A new study with a different design, including only the highest risk areas, is recommended to better analyze the contribution of socioeconomic variables as possible drivers. A previous study suggested the importance of socioeconomic status in leptospirosis infection even after controlling the environmental factors [51]. If similar environmental drivers were analyzed separately, it could be possible that the socioeconomic variability in the risk area could be more visible. In rural Nicaragua, it is common to use creeks for washing clothes and bathing all in the same location where animals come to drink water. These behaviors probably increase the risk. However, a different design is needed to better analyze these risk factors.

In the literature review, we found no study analyzing the risk of leptospirosis in the entire country and exploring possible drivers at an ecological level using the available secondary data from the country’s surveillance system. A recent study in American Samoa was developed analyzing the possible environmental drivers of leptospirosis for the entire country (around 67,000 populations) through a cross-sectional seroprevalence study [52,53]. They found a significant association with living in lower altitudes. In our study, we analyzed the percentage of municipal terrain with flat to moderate slope (25% or less), and it was not possible to suggest such an association at this level of aggregation. In the Samoan study, a significant association with piggeries was also found, while in our study no association was suggested from the analysis of the entire nation by municipalities.

The limitations of ecological studies, in this case by municipality, are well known. More disaggregated studies are suggested to evaluate risk factors. However the purpose of this study was to support interventions in the countries that are usually structured geopolitically in this way. Identifying critical areas and possible drivers in Nicaragua could not only support a review of their Intersectoral Plan, but also more importantly establish a methodology to support other countries in the identification of their risk areas even where the surveillance of leptospirosis is not strongly in place.

The use of mapping techniques, specifically GIS, is recommended for the application of research results in public health evidence-based decision making. Through the use of GIS, indicators are gathered from different sources and placed in a common database for statistical and geographical analysis [54].

To confirm that Leon and Chinandega departments and adjacent municipalities are a clustered geographical region where the concentration of leptospirosis cases is in excess of the endemic rate, other spatial analysis techniques could be used to identify hotspots, such as provided by Kulldorff or Getis-Ord Gi * as well as the Moran-Anselin autocorrelation analysis, available in ArcGIS 10.0. Using this simple risk stratification, however, it was also possible to demonstrate that these two departments are the ones with higher percentages of critical areas (Table 3).

This study, along with the further analyses recommended above, could be part of an evidence-based approach to health planning, a stepping stone to sharing methodological experiences between the countries of Central America. They could represent the bases for a capacity development project in the subregion, or to cite the UNDP capacity development approach “a real-world application to strengthen and contribute to national capacities for development” [55].
